# Reviving the Rarity of Cecal Lymphangioma: A Case Report

**DOI:** 10.7759/cureus.69209

**Published:** 2024-09-11

**Authors:** David A Eppley, Zaid Rana

**Affiliations:** 1 Internal Medicine, Lake Erie College of Osteopathic Medicine, Fort Lauderdale, USA; 2 Gastroenterology, Keralty Hospital, South Miami, USA

**Keywords:** routine colonoscopy finding, benign cecal neoplasm, incidental cecal lesion, rare gastrointestinal tumor, colonoscopic removal, colonic lymphangioma, adult lymphangioma

## Abstract

The present case report describes the incidental discovery of cecal lymphangioma, a rare benign neoplasm originating from malformations of lymphatic vessels. Lymphangiomas are uncommon in the gastrointestinal tract, and their presence in the colon is particularly unusual. This finding adds to the limited literature on colonic lymphangiomas and emphasizes recognizing these unusual lesions. Further research is needed to understand better their clinical characteristics, potential complications, and optimal management strategies, especially in atypical locations such as the colon.

## Introduction

Lymphangiomas are benign neoplasms that result from malformations of the lymphatic vessels, leading to abnormal collections of lymphatic fluid within cystic spaces [1]. These exceedingly rare tumors have an estimated incidence ranging from 1 in every 27,000 to 1 in 250,000 individuals [1,2]. Approximately 95% of these lesions occur in the head, neck, and axilla, with only 5% found in other locations, such as the mesentery, retroperitoneum, lung, mediastinum, and colon [3-5].

The pathophysiology of lymphangiomas is not fully understood but is believed to involve lymphatic obstruction, traumatic stimuli, congenital absence of lymphatic channels, general aging of the bowel, or radiation exposure [3,6]. While lymphangiomas are benign, their presence in atypical locations like the colon can present diagnostic challenges and potential complications. Given the rarity of colonic lymphangiomas and the limited documentation in the literature, every new case provides valuable insight into the clinical characteristics, diagnosis, and management of this unusual entity.

With the recent reduction of the recommended screening age from 50 to 45, the frequency of colorectal cancer screenings is increasing, leading gastroenterologists to encounter a wider variety of colonic lesions, including rare conditions like lymphangiomas [7]. This case report seeks to address the gap in the literature by offering a detailed account of a cecal lymphangioma discovered during routine colonoscopy, thus enhancing clinicians' understanding of this extraordinary condition and guiding appropriate management strategies.

## Case presentation

A 56-year-old nulligravida, Hispanic woman with no significant past medical history presented for her first routine screening colonoscopy. The patient denied abdominal pain, weight loss, bloody stool, or changes in bowel habits. She also denied a family history of colorectal cancer, polyps, or inflammatory bowel disease.

During the colonoscopy, a single small polyp, measuring 0.1 x 0.1 x 0.1 cm, was discovered on the wall of the cecum and fully resected using cold snare polypectomy. No other abnormalities were detected during the procedure, and the patient recovered uneventfully.

**Figure 1:**
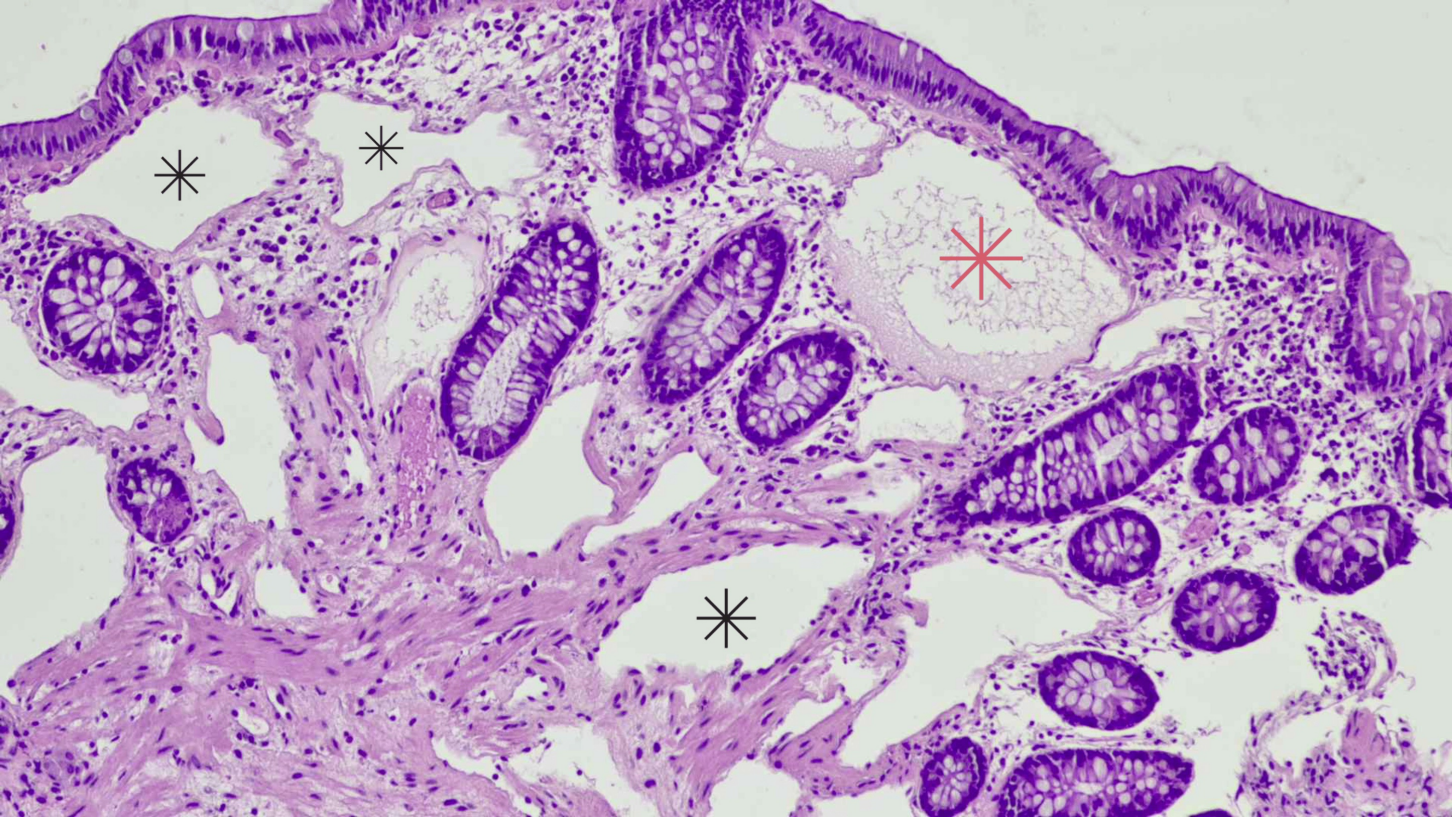
Pathology sample of lymphangioma with hematoxylin and eosin stain, magnification 100x Black asterisk indicates dilated lymphatic channels. Red asterisk indicates dilated lymphatic channels with fluid present. Typical cecal glands and surrounding stroma are visible.

**Figure 2:**
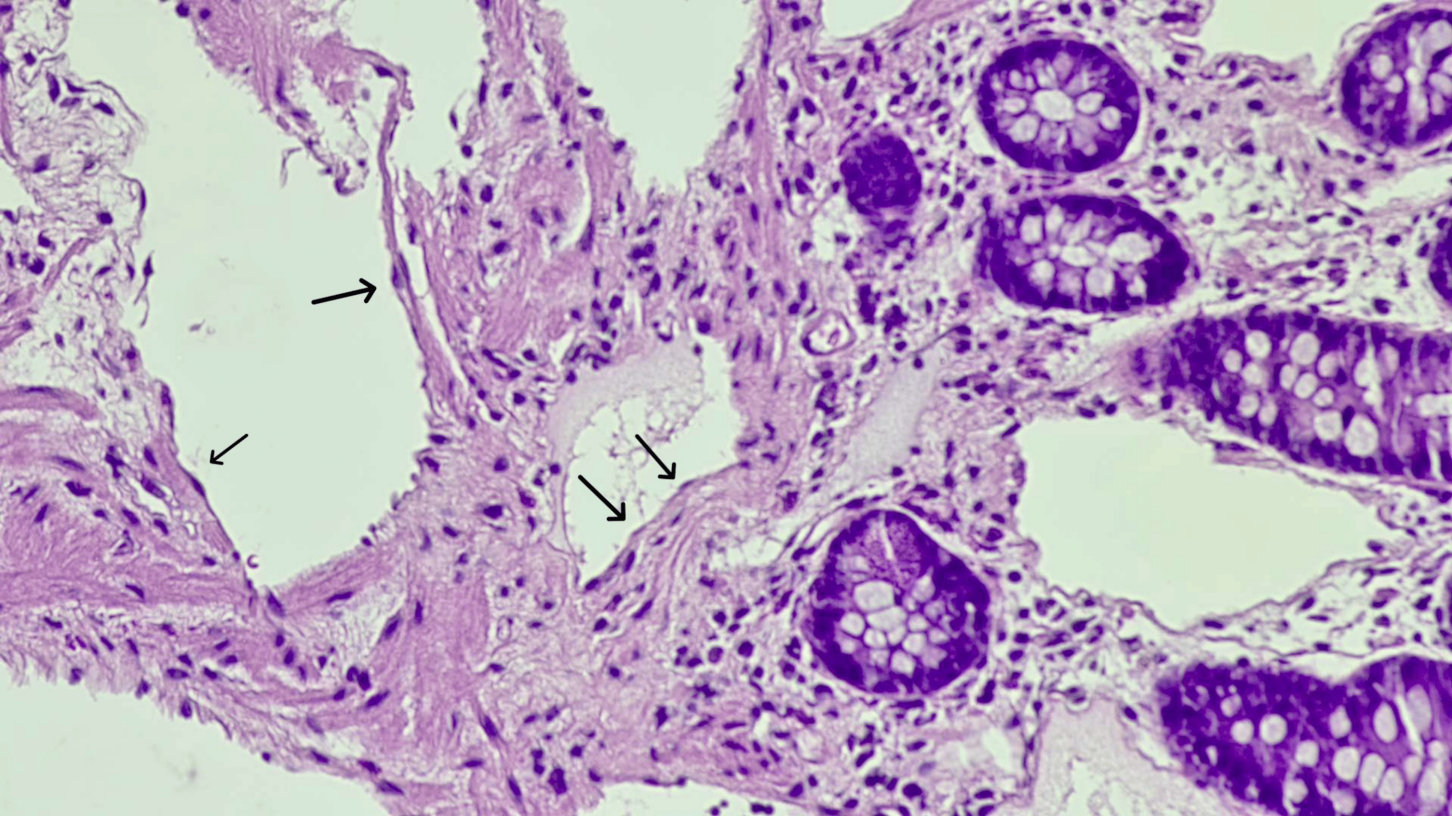
Pathology sample of lymphangioma with hematoxylin and eosin stain, magnification 200x Arrows indicate endothelial cells lining lymphatic channels.

Histopathological examination of the polyp revealed multiple large, dilated lymphatic channels filled with lymphatic fluid, with an absence of red blood cells, further indicating the lymphatic origin of the vessels (Figure 1). The channels were lined by a single layer of endothelial cells, a type of simple squamous epithelial cell, confirming the diagnosis of cecal lymphangioma (Figure 2).

## Discussion

As previously mentioned, lymphangiomas in the gastrointestinal tract are especially rare, with even fewer reported cases involving the colon. A study investigating abdominal lymphangiomas over 22 years identified 107 cases, of which only eight were colonic, highlighting the rarity of this condition in the colon [3].

Histopathologically, lymphangiomas are characterized by multiple dilated lymphatic channels lined by a single layer of endothelial cells, as observed in this case [4,8]. These channels may form interconnecting cysts filled with lymphatic fluid, which can flow into adjacent channels, as seen in our specimen (Figure 1) [8,9].

Small lymphangiomas located within the colon, such as the one presented in this case, can often be successfully resected endoscopically using snare techniques or ligation loops [8]. Endoscopic resection is minimally invasive and typically sufficient for small, isolated lesions [8,10]. However, larger lymphangiomas, or those extending beyond the colon, may require laparoscopic resection due to the risk of perforation with endoscopic techniques or if the lesion is inaccessible via endoscopy [8].

The incidental finding of this lymphangioma highlights the importance of routine colonoscopy, not only for the detection of precancerous polyps but also for identifying other rare lesions that may have significant clinical implications if left untreated. Although lymphangiomas are benign, their potential to grow large enough to cause complications, such as bowel obstruction or intussusception, cannot be overlooked [11,12]. Adult intussusceptions are typically caused by lipomas or adenomas, but lymphangiomas have accounted for 5-10% of adult intussusceptions [11,12].

## Conclusions

In conclusion, this case emphasizes the extraordinary rarity of colonic lymphangiomas, particularly in the cecum, where their occurrence is exceptionally uncommon. The incidental discovery of this benign lesion during a routine colonoscopy highlights the unusual nature of such findings in the gastrointestinal tract. Given the scarcity of documented cases, each new report adds valuable insight into the clinical understanding of these unexpected entities, reinforcing the significance of routine screenings in uncovering not just common conditions but also rare and unexpected lesions.
